# Avirulence of a spontaneous *Francisella tularensis* subsp. *mediasiatica prmA* mutant

**DOI:** 10.1371/journal.pone.0305569

**Published:** 2024-06-18

**Authors:** Vitalii Timofeev, Irina Bakhteeva, Galina Titareva, Raisa Mironova, Vera Evseeva, Tatiana Kravchenko, Angelika Sizova, Alexander Borzilov, Natalia Pavlovich, Alexander Mokrievich, Ivan Dyatlov, Gilles Vergnaud

**Affiliations:** 1 State Research Center for Applied Microbiology and Biotechnology (SRCAMB), Obolensk, Russia; 2 Rostov-on-Don Antiplague Institute, Rostov-on-Don, Russia; 3 CEA, CNRS, Institute for Integrative Biology of the Cell (I2BC), Université Paris-Saclay, Gif-sur-Yvette, France; University of Rochester, UNITED STATES

## Abstract

*Francisella tularensis*, the causative agent of tularemia, is divided into three subspecies. Two of these, subspecies *holarctica* and *tularensis*, are highly pathogenic to humans and consequently relatively well studied. The third subspecies, *mediasiatica*, is rarely isolated and remains poorly studied. It is distributed in the sparsely populated regions of Central Asia and Siberia. Curently this subspecies is not known to have been responsible for human infections in spite of its high virulence in laboratory animals. Subspecies *mediasiatica* is currently divided into three subgroups—MI, present in Central Asia, MII, present in southern Siberia, and MIII represented by a unique strain, 60(B)57, isolated in Uzbekistan in 1960. We describe here the unexpected observation that MIII strain 60(B)57 is avirulent and immunogenic. We observed that infection with this strain protected mice from challenge 21 days later with a virulent subsp. *mediasiatica* strain. With an increase of this interval, the protection for mice was significantly reduced. In contrast, guinea pigs were protected from challenge with strains of the subspecies *holarctica* and *mediasiatica* (but not subsp. *tularensis*) 90 days after infection with 60(B)57. We performed genome assembly based on whole genome sequencing data obtained using the Nanopore MinION for strain 60(B)57 and two subsp. *mediasiatica* strains representing the Central Asian MI and Siberian MII phylogenetic subgroups. The *prmA* gene is truncated due to a nonsense mutation in strain 60(B)57. The deletion of gene *prmA* has previously been shown to induce a loss of virulence in *Francisella novicida* the closest model organism suggesting that the observed mutation might the cause of the avirulence of strain 60(B)57.

## Introduction

*Francisella tularensis* is a small Gram-negative aerobic coccobacillus. It is a facultative intracellular parasite capable of infecting a wide range of animals and causing a plague-like disease called tularemia. *F*. *tularensis* is currently divided into three subspecies—*holarctica*, *tularensis*, and *mediasiatica* [1, 2]. *Francisella novicida* sometimes considered as a fourth non-pathogenic subspecies is the nearest neighbor and is used as model organism, not subjected to the strict regulations applying to the manipulation of *F*. *tularensis* [1–3]. Two *F*. *tularensis* subspecies are known to be pathogenic for humans, subsp. *tularensis* present in North America, and subsp. *holarctica* common throughout the Northern Hemisphere [4, 5]. Due to epidemiological significance and to the distribution area, including countries with a developed sanitary supervision system, these two subspecies are relatively well studied compared to the third subspecies, *mediasiatica*. This subspecies was initially detected in some regions of Kazakhstan and Turkmenistan in Central Asia.

Over the past decade, we reported that subsp. *mediasiatica* is significantly more geographically widespread than previously established. Subsp *mediasiatica* strains were found in a natural focus of tularemia in Altai (Russia) and more recently the same *mediasiatica* lineage was recovered in Siberia a few hundred kilometers east of the Altai focus [6, 7].

Genetic analysis of subsp *mediasiatica* strains of different geographical origin allowed to divide the subspecies into three very closely related subgroups: MI, represented by "classic" strains from Central Asia; MII, combining Altai and Siberian strains, and MIII. MIII subgroup is currently represented by only one strain 60(B)57 isolated in Karakalpakstan, Uzbekistan in 1960 [6, 7].

Virulence studies have shown that although subsp. *mediasiatica* has not so far been associated with human cases of tularemia, this subspecies is virulent for laboratory animals. More specifically, it is less virulent than subsp. *tularensis*, but more virulent than subsp. *holarctica* [6, 8]. Published information indicated that subsp. *mediasiatica* is as virulent in rabbits as subsp. *holarctica* [2]. These experiments used MI and/or MII strains. We evaluated here the virulence of MIII strain 60(B)57 maintained by the Rostov-on-Don Anti-Plague Institute. We were surprised to find that strain 60(B)57 was not able to cause the death of mice during experimental infection. The infection induced an immune response that protected animals from subsequent infection with a virulent *F*. *tularensis* strain. In the present report we describe the results obtained, including the full genome sequencing obtained by long sequencing reads of one mediasiatica from each of the three lineages, MI, MII and MIII.

## Materials and methods

### Strains

Three *F*. *tularensis* subsp. *mediasiatica* strains were used, strain 60(B)57 (subgroup MIII), strain A-678 (subgroup MII, isolated in Altai), known to be virulent in animals (LD_50_ for mice <5 CFU, LD_50_ for guinea pigs <50 CFU), and strain 120 (subgroup MI). We also used two virulent strains of other subspecies—subsp. *holarctica* strain 503 and subsp. *tularensis* strain SCHU S4 in animal experiments.

### Bacterial cultures

The strains were grown at 37 °C on solid (FT-agar) and liquid (FT-broth) nutrient media (SRCAMB, Obolensk, Russia).

### Animal experiments

#### Ethics statement

All protocols for animal experiments were approved by the State Research Center for Applied Microbiology and Biotechnology Bioethics Committee (Permit No: VP-2023/2). They were performed in compliance with NIH Animal Welfare Insurance #A5476-01 issued on 02/07/2007 and the European Union guidelines and regulations on the handling, care, and protection of laboratory animals (https://eur-lex.europa.eu/eli/dir/2010/63/oj). A minimum number of animals was used for the experiments. The approved protocols provided scientifically validated humane endpoints, including pre-set criteria for the euthanasia of moribund animals via CO_2_ inhalation. Animals were euthanized when they became lethargic, dehydrated, moribund, unable to rise, or non-responsive to touch. Health conditions were monitored at least twice a day throughout the entire period between the challenge and the end of the experiment, which was 21 days. All animals reaching endpoint criteria were euthanized immediately. All manipulations with animals were carried out by employees who underwent specialized training.

#### Animals

BALB/c mice (5–8 weeks-old, 18–20 g) and guinea pigs (5–8 weeks-old, 275 ± 25 g) of both genders, purchased from the Laboratory Animals Breeding Center Shemyakin and Ovchinnikov Institute of Bioorganic Chemistry, Russia, were used in the experiments. In total, we used 69 mice (29 died, eight of which were euthanized) and nine guinea pigs (three died, two of which were euthanized).

Animals were housed in polycarbonate cages with space for comfortable movement (five mice in a 484 cm^2^-cage and three guinea pigs in a 864 cm^2^-cage) with ad libitum access to food (Mouse Mixed Fodder PK-120, and Guinea Pig Mixed Fodder КК–122, Laboratorkorm, Russia) and tap water, under constant temperature and humidity conditions (22 °C ± 2 °C and 50% ± 10%, respectively) and a 12-h light/12-h dark cycle.

#### Virulence and immunogenicity studies

BALB/c mice were inoculated subcutaneously with 0.1 mL *F*. *tularensis* cells in PBS in the inner part of the upper thigh. Infection and challenge doses are indicated in the "Results" section.

### Statistics

The obtained data were statistically processed using the GraphPad Prism 7 program (https://www.graphpad.com/). The data were presented as the means ± standard error of the mean (SEM). One-way ANOVA (Mann–Whitney), two-way ANOVA (Dunnett’s multiple comparisons), and nonparametric (Kolmogorov–Smirnov and Kruskal–Wallis) tests were used to compare the weight and lifespan data. A log-rank test, a log-rank test for trends and a Gehan–Breslow–Wilcoxon test were used to compare survival curves. P values less than 0.05 were considered significant.

### Whole-Genome Sequencing (WGS) and assembly

Whole genome sequencing was completed using the Oxford Nanopore MinION following the manufacturer’s protocols. A rapid barcoding kit (RBK004) and MinION flow cell (R9.4.1) were used for long read sequencing (Oxford Nanopore Technologies). Data aquisition was performed by running MinKNOW software v18.05.5 (time, 48 h; 180 mV), and base calling was performed using Albacore v2.3.1 (Oxford Nanopore Technologies) with default settings.

Flye version 2.7.1 was used with default parameters to assemble the long reads sequence data [9]. Assemblies were annotated using the DFAST pipeline version 1.2.20 [10].

The Mauve plugin from GeniousPrime version 2019.2.3 (Biomatters, New Zealand) was used for pairwise comparisons of assemblies [11]. Whole chromosome comparisons for mutations search were run using the chromosome comparisons tool within BioNumerics version 8.1, with default parameters. Candidate nonsense mutations were checked by analysing previously published short reads sequence data using GeniousPrime version 2019.2.3 (bioproject PRJNA870100, sequence read archive SRR21146809) [7, 12].

#### Study of comparative ability to reproduce in macrophages

Mouse macrophage-like cells of the J774.A1 line at a concentration of 2–5×10^5^ cells/ml were cultured in DMEM medium supplemented with 10% inactivated fetal calf serum and 2 mM glutamine in 24-well plates within 24 hours for conditioning. A bacterial suspension of *F*. *tularensis* in DMEM medium was added to the J774.A1 monolayer with a ratio of 100 bacterial cells per macrophage. After this, the plate was incubated at 37 °C and 5% carbon dioxide for 3 hours, then treated for one hour with gentamicin (10 mg/l) to destroy non-phagocytosed bacteria. After washing three times to remove the antibiotic, the J774.A1 monolayer was lysed with distilled water in a fraction of the wells and tenfold dilutions of the lysate were plated onto FT agar plates. Macrophages in the remaining wells continued to be cultured in DMEM supplemented with 10% fetal calf serum for 24 hours at 37°C and 5% carbon dioxide, after which they were also lysed with distilled water and dilutions were plated in strips on FT agar plates. After 2–3 days, the number of CFU was counted.

#### RT-PCR

Total RNA was extracted using “RNA-EXTRAN” kit (Syntol, Moscow, Russia) according to the manufacturer’s instructions. cDNA was synthesized using the “REVERTA-L” kit (AmplySens, Moscow, Russia). SYBR Green based quantitative PCR was performed using“2.5-fold PCR mixture with SYBR-Green” kit (Syntol, Moscow, Russia) with a CFX96 real-time PCR system (BIO-RAD). The following primers were used: iglCF (ACAGGTAACAAGTGGCGAGACC), iglCR (AAACACCCATAAGTTCTGTTGGCTC), 16SF (AGAGTTTGATCCTGGCTCAG) and 16SR (GTATTACCGCGGCTGCTG). We used the following amplification program: denaturation 95°C 5 minutes then 40 cycles 95°C 30 sec, 56°C 30 sec, 72°C 30 sec + plate read.

## Results

### Origin of strain 60(B)57

Strain 60(B)57 was isolated in Nukus anti-plague station from *Hyaloma* sp. ticks collected in the Chimbay region, Karakalpakstan, Uzbekistan in 1960. Since anti-plague stations do not have the capacity to store cultures of pathogenic microorganisms after their identification, the strain was transferred to the Central Asian Research Anti-Plague Institute (currently “Kazakh Scientific Center of Quarantine and Zoonotic Diseases”, KSCQZD), located in Almaty, Kazakhstan. The Rostov-on-Don Antiplague Institute received the strain from KSCQZD in 1986. In all our studies we used ampoules with freeze-dried culture deposited in 1986. Therefore, the 60(B)57 strain has not been passaged many times since 1986. In the passport of the strain, which came along in 1986 with the strain itself from KSCQZD, nothing was indicated in the column "virulence for laboratory animals" and we could not find information regarding the estimation of the virulence of strain 60(B)57 in Uzbekistan, Kazakhstan or at the Rostov-on-Don Antiplague Institute.

### Virulence determination and evaluation of immunogenic potential

In 2022, strain 60(B)57 previously shown to define *mediasiatica* lineage MIII based on DNA analysis [6] was transferred from the Rostov-on-Don Anti-Plague Institute to SRCAMB in the form of a live culture. As part of the standard protocol for deposision of a new strain in the SRCAMB collection, the virulence of the strain was checked. Surprisingly given the behaviour of other *mediasiatica* strains, three mice, infected with 100 CFU/animal survived without showing any signs of the disease (decreased mobility, refusal to eat, matted fur). After euthanasia, strain 60(B)57 could be cultured from the spleens of mice 10 days after infection. Assuming that we could face a decrease in the ability of the strain to survive in vivo, which sometimes occurs with a long laboratory maintenance of pathogens, we carried out the so -called "animalization" of the strain. This consists in three consecutive cycles of infection—euthanasia—strain cultivation at the infection dose of 100 CFU/animal. In a number of cases, according to our experience, this approach made it possible to select pathogen cells that have an increased ability to survive in the host organism. The derived strain that underwent this procedure, subsequently named 60(B)57A, did not change its virulence properties, and was still not able to cause a lethal infection in mice at a dosage of 100 CFU/animal.

Thus, we suspected that the virulence of strain 60(B)57 is significantly different from the virulence of all natural *F*. *tularensis* and *F*. *novicida* strains in our collection. To study this issue in more details, we conducted a larger experiment with both strains, 60(B)57 and derived 60(B)57A. BALB/c mice (five per group) were infected at doses ranging from 10^2^ to 10^8^ CFU with a step of one order for a total of seven groups and 35 mice for each strain. Since all mice survived the infection, the LD_50_ value could not be determined. During this experiment, we also assessed the state of mice by assessing body weight. The dynamics of body weight is shown in S1 Fig. The groups infected with strain 60(B)57 showed no decrease in body weight. The groups infected with high doses (from 10^6^ to 10^8^) of strain 60(В)57А showed a decrease in body weight after infection and a delay in weight gain for several days. This effect was dose-dependent, that is, it increased with increasing infecting dose. We did not find other signs of morbidity, such as necrotic changes at the injection site or decrease in mobility. Thus, we can state that strain 60(B)57 is completely avirulent in mice. It is not capable of causing a lethal or even serious disease, including at extremely high infecting doses, although it belongs taxonomically to the virulent subspecies. At the same time, its cells are able to persist in the host organism. Strain 60(B)57 is similar in these properties to live vaccine strains, such as 15NIIEG and the derived LVS. We next evaluated how much the 60(B)57 strain is similar to vaccine strains in the ability to protect against subsequent infection with virulent strains.

**Fig 1:**
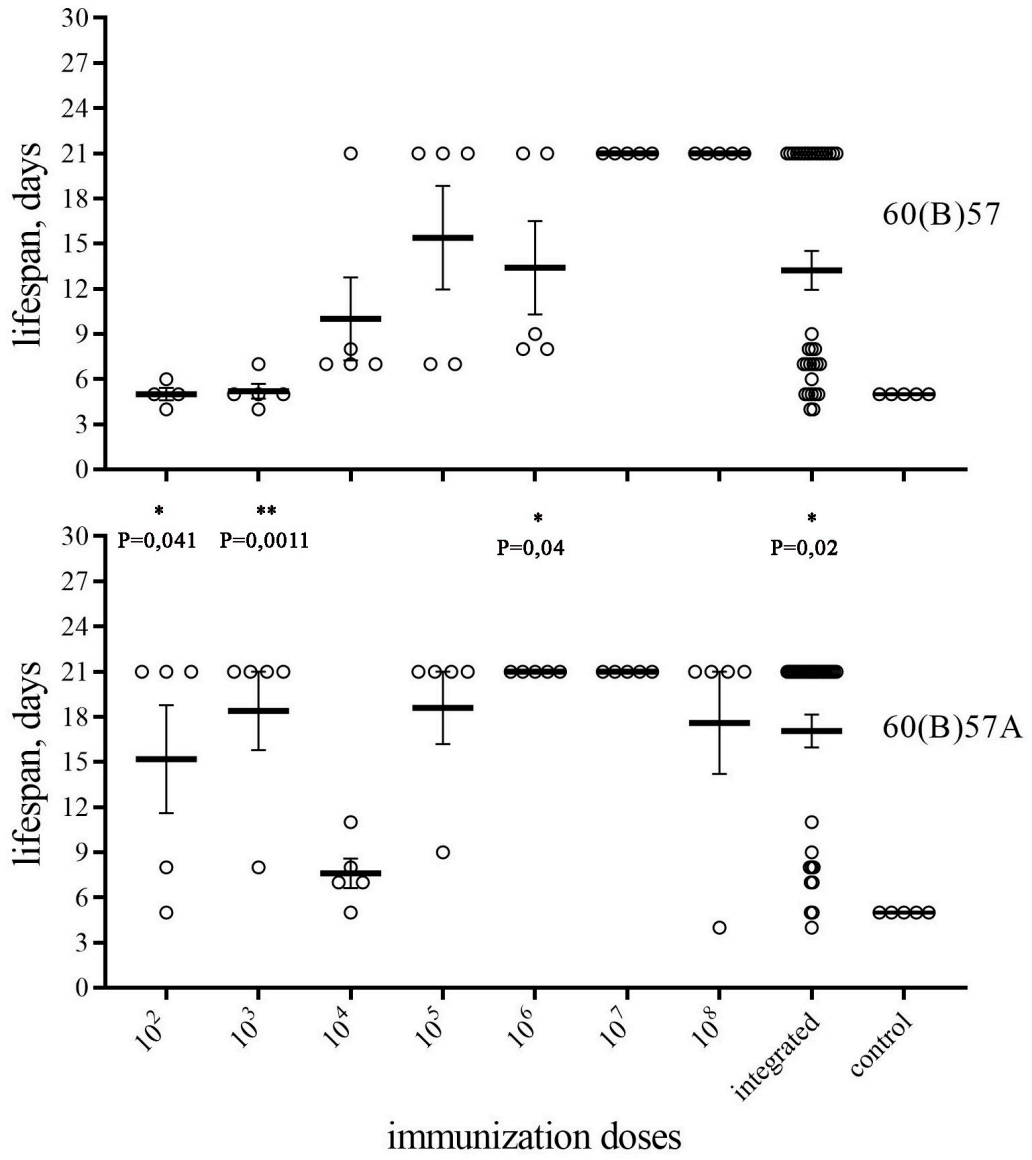
Lifespan of mice infected with strains 60(B)57 (upper part) and 60(B)57A (lower part) after subsequent challenge with virulent subsp.*mediasiatica* strain A-678. For each infection dose, the lifespan of the five mice is indicated. Each part of the figure also includes integrated data for the 35 mice infected with these strains, regardless of the size of the infecting dose The time interval between infection and challenge is 21 days [13]. Each circle represents an individual animal. A lifespan of 21 days means survival of the animal during the entire post-challenge observation period of 21 days. Bold horizontal lines indicate the average values of the lifespan of the group, values are indicated with the standard error of the mean (SEM). The average lifespan was calculated taking into account the surviving mice (21 days). The same control group of naive mice is depicted in both parts of the figure. Statistically significant differences between groups infected with the same dose are indicated with a p value calculated using Unpaired t test.

For this purpose, we used animals that were infected with strains 60(B)57 and 60(B)57A in the previous experiment. 21 days after infection with avirulent strains, we challenged all survived mice and a control group of five naive mice with virulent subsp. *mediasiatica* strain A-678 at a dosage of 1000 CFU/animal. The results obtained are shown in Fig 1 in the form of graphs of the lifespan of infected animals.

The obtained data indicate that a high infection dose (10^7^−10^8^ cfu) with either strain provided protection against subsequent challenge with *mediasiatica*. The results also suggest that infection with strain 60(B)57A more effectively protects mice than infection with the original strain 60(B)57 at the lower infection doses. This effect is expressed both at the lower doses (10^2^ and 10^5^, respectively), which protected most of the infected animals, and at the higher doses (10^6^ and 10^7^, respectively), which protected the entire infected group.

These observations suggested that the 60(B)57 strain is not only avirulent, but also has some protective properties. Consequently we compared this strain with the existing live vaccine, *F*. *tularensis* subsp. *holarctica* 15NIIEG. In this experiment, we increased the time interval between infection and challenge to 90 days. We chose a period of 90 days based on our previously published results [8], according to which, starting from the 90th day after vaccination of mice and guinea pigs with strain 15NIIEG, strains of different subspecies begin to show differences in their ability to overcome post-vaccination immunity. At a shorter time interval (30 and 60 days), we did not observe such an effect. We infected mice and guinea pigs with 60(B)57A and 15NIIEG strains at the following dosages: 10^6^ CFU 60(В)57A/animal and 50 CFU 15NIIEG/animal. 90 days after infection, the animals were challenged subcutaneously with *F*. *tularensis* strains belonging to each of the three subspecies SCHU S4 (subsp. *tularensis*), 503 (subsp. *holarctica*) and A-678 (subsp. *mediasiatica*). The challenge dose was 10^3^ CFU/animal. The number of animals in each group is presented in Table 1. The animals were observed for 21 days after challenge.

**Table 1:** The number of animals in groups infected with strains 15NIIEG and 60(B)57A and subsequently challenged with strains SCHU S4 (subsp. *tularensis*), 503 (subsp. *holarctica*) and A-678 (subsp. *mediasiatica*). The time interval between infection and challenge is 90 days.

Animal species	Infection strain	Challenge strain	Number of animals in the group	Number of death
mice	15NIIEG	503	6	0
		A-678	7	1
		SCHU S4	6	0
	60(В)57А	503	10	9
		A-678	10	9
		SCHU S4	10	9
	naive (control)	503	3	3
		A-678	3	3
		SCHU S4	3	3
guinea pigs	15NIIEG	503	3	0
		A-678	3	0
		SCHU S4	3	0
	60(В)57А	503	3	0
		A-678	3	0
		SCHU S4	3	3
	naive (control)	503	3	3
		A-678	3	3
		SCHU S4	3	3

The time interval between infection and challenge is 90 days.

**Fig 2:**
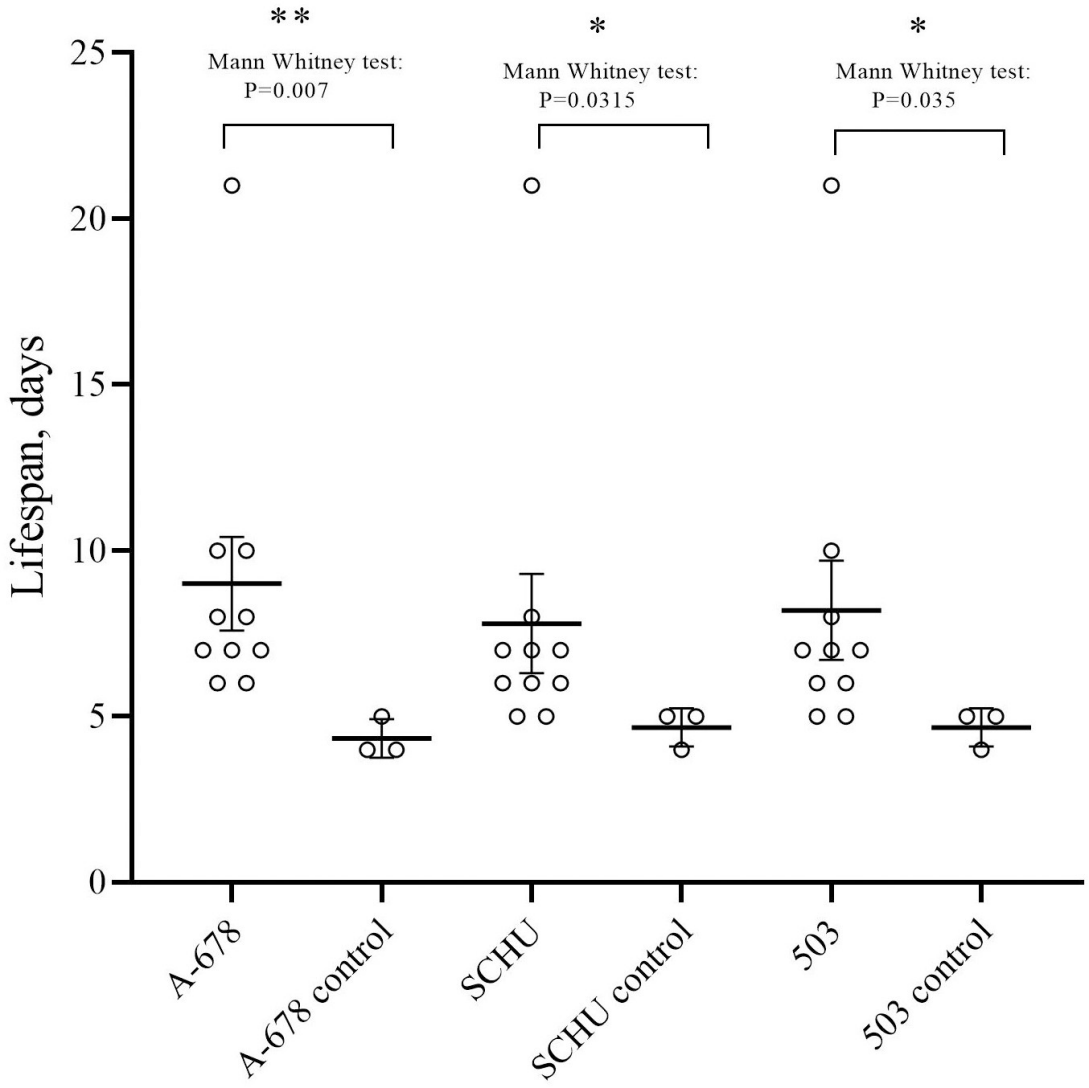
Lifespan of mice infected with strain 60(B)57A (10^6^ CFU/animal) or not and challenged with strains subsp. *tularensis* SCHU S4, subsp. *holarctica* 503 or subsp. *mediasiatica* A-678. The time interval between infection and challenge is 90 days. Lifespan values indicated with SEM. Statistically significant differences in survival between the infected and control groups are indicated. No statistically significant differences in survival were found between the infected groups.

Almost all mice infected with vaccine strain 15NIIEG survived the challenge. We observed the death (4^th^ day) of one mouse challenged with subsp. *mediasiatica* strain A-678. The control mice all died on the 4th-5th day. Strain 60(B)57A provided almost no protection, 9 out of 10 mice died in each group. The mean time to death (in days) was 7.8±1.5 for strain SCHU S4, 8.2±1.49 for strain 503, and 9±1.4 for strain A-678 (Fig 2).

**Fig 3:**
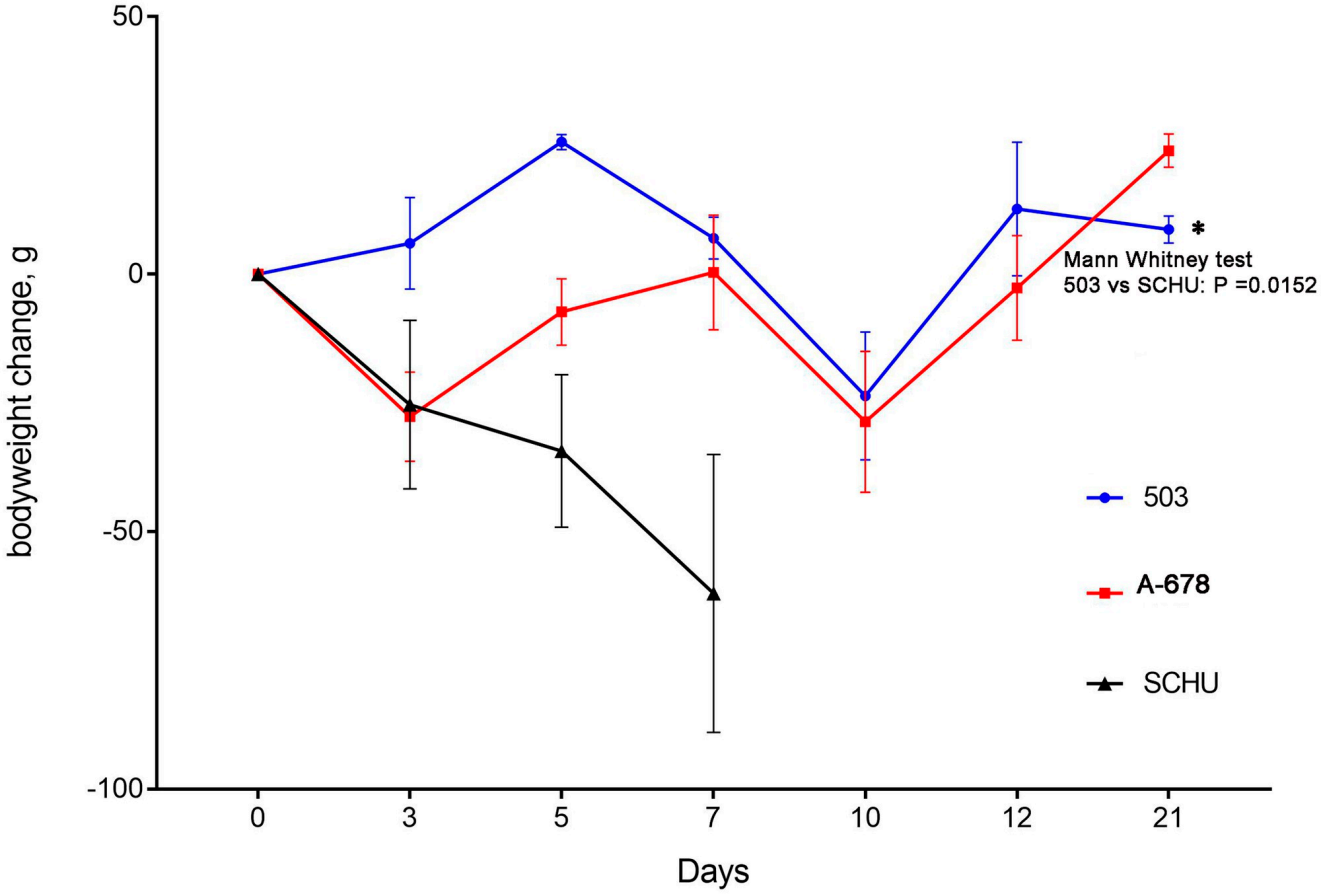
Dynamics of average bodyweight of guinea pigs infected with strain 60(B)57A (10^6^ CFU/animal) and challenged with virulent strains: Subsp. *tularensis* SCHU S4, subsp. *holarctica* 503 or subsp. *mediasiatica* A-678. The time interval between infection and challenge is 90 days. Values are indicated with SEM. A statistically significant difference between the 503 and SCHU groups is indicated by the figure.

All guinea pigs infected with vaccine strain 15NIIEG survived the challenge by all three strains (Fig 1). Guinea pigs vaccinated with strain 60(B)57A survived challenge with *holarctica* strain 503 and *mediasiatica* strain A-678, but not with *tularensis* strain SCHU S4. The three animals died on days 6, 8 and 10. All control guinea pigs died on day 6 or 7. In addition to accounting for mortality, we measured the dynamics of the bodyweight of guinea pigs infected with the strain 60(B)57A and challenged with virulent strains. We found that although the guinea pigs survived challenge with strains of the *holarctica* and *mediasiatica* subspecies, they lost weight (Fig 3), indicating that an infection process was taking place. In this experiment, we did not weigh the guinea pigs infected with the vaccine strain 15NIIEG. However, according to the data we obtained earlier [8], the bodyweight loss of guinea pigs after vaccination with this strain and subsequent infection 90 days later is less pronounced. No decrease in bodyweight was observed in the case of challenge with a subsp. *holarctica* strain. Thus, strain 60(B)57A turned out to be much less protective for mice and guinea pigs than strain 15NIIEG, especially considering the much higher infecting dose that we used.

In addition to the mouse model, we used a mouse macrophage-like cell model to evaluate the comparative virulence of strain 60(P)57A. The ability of the *F*. *tularensis* strain to infect macrophages, multiply in them and lyse them to a certain extent correlates with the virulence of this strain in animals. In this experiment we compared strain 60(P)57A with vaccine strain 15NIIEG. Reproduction efficiency was assessed by the difference between the decimal logarithms of CFU obtained from inoculating macrophage lysates 3 and 48 hours after infection with the compared *F*. *tularensis* strains. We found that strain 60(P)57A had almost lost the ability to proliferate within murine macrophage-like J774.1A cells (Table 2).

**Table 2:** Reproduction of strains 60(B)57A and 15 NIIEG in macrophages of the J774.A1 line. Results are presented in CFU/ml (mean with SEM).

	Time of phagocytosis	Strain	
		15 NIIEG	60(B)57А
CFU/ml	3 h	4,7×10^3^ ± 9,2×10^2^	3,4×10^3^ ± 4,2×10^2^
	24 h	1,3×10^6^ ± 1,4×10^5^	3,7×10^3^ ± 7,8×10^2^
Intracellular proliferation index		276,6	1,1

### Whole genome sequencing and chromosome sequence reconstruction

In order to try and identify the genetic cause of the absence of virulence of strain 60(B)57, we sequenced its DNA, as well as the DNA of subsp. *mediasiatica* group MI strain 120 and subsp. *mediasiatica* group MII strain A-678 using Oxford Nanopore Technologies. Raw data accession numbers are SRR28129393 for strain 60(B)57A, SRR28129394 for strain 120 and for strain SRR28129395 A-678. The resulting long reads could be successfully assembled in a single contig. The reconstructed chromosome sequences for strains 60(B)57, 120, and A-678 were deposited in GenBank, assembly accession numbers are listed in S1 Table.

**Fig 4:**
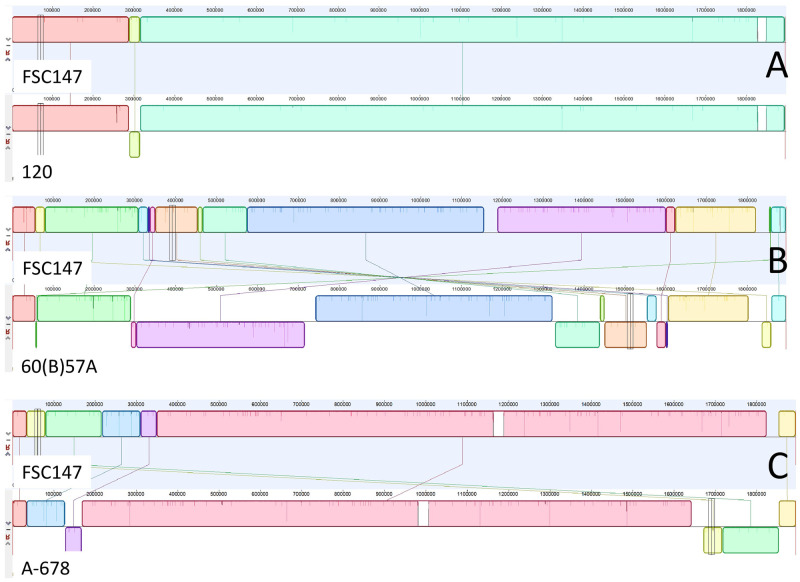
Genome alignment of subsp. *mediasiatica* MI strain FSC147 with each of the three *mediasiatica* strains 120, 60(B)57 and A-678. Panel A: alignment of FSC147 (assembly accession GCA_000018925.1) with strain 120. Both strains belong to the MI lineage. The genomes are almost colinear, and differ by a single sequence inversion of approximately 26 kilobases flanked by IS elements. Panel B: alignment of FSC147 from *mediasiatica* lineage MI with 60(B)57A from lineage MIII. Fifteen synteny blocks are detected. Panel C: alignment of FSC147 from *mediasiatica* lineage MI with A-678 from lineage MII. Seven synteny blocks are detected.

**Fig 5:**
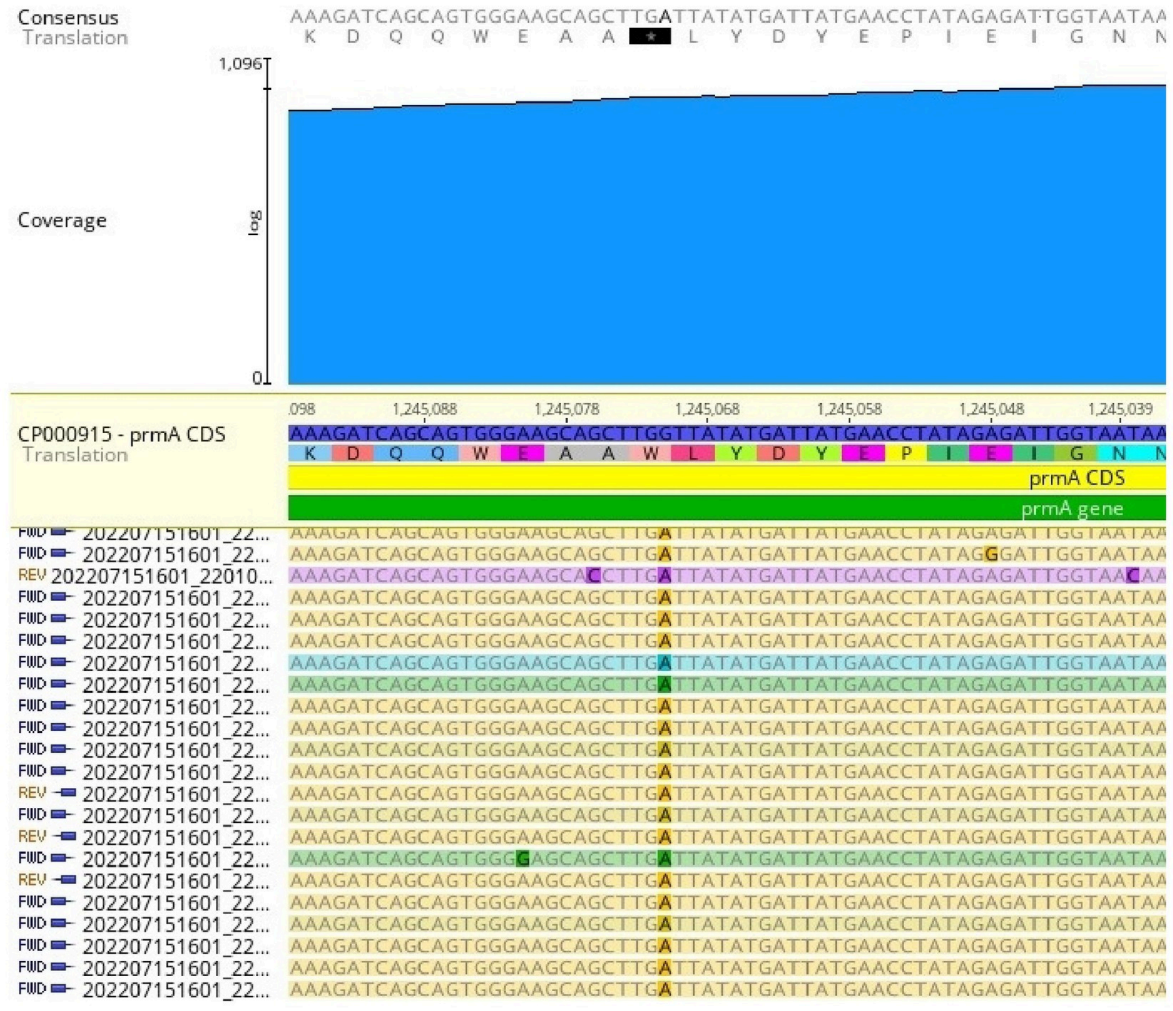
Mapping of the 60(B)57 short reads on FSC147 *prmA* gene. Previously published short reads from sequence reads archive SRR21146809 [7] were mapped on the *prmA* gene to confirm the sequence derived from long reads. Position 1,245,071 is A instead of G in all 444 reads mapping on that position, causing a nonsense mutation within *prmA*.

We then compared the 60(B)57A genome assembly (accession GCA_030505555.1) with assembly accession GCA_000018925 (*mediasiatica* lineage MI strain FSC147) in addition to strains 120 (accession GCA_030505515.1) and A-678 (accession GCA_030505475.1) [1]. The 60(B)57A genome differed from the others by a few large scale rearrangements as well as small scale rearrangements (tandem repeats) in agreement with previous observations (Fig 4) and single nucleotide mutations. Most of these changes are predicted to have a low impact (point mutations in intergenic regions, synonymous changes in coding regions). No differences could be observed in the *F*. *tularensis* pathogenicity island [14, 15]. S2 Table provides the list of 130 nonsynonymous mutations which could be identified. The list includes three nonsense mutations. The first one at position 345,966 in the FSC147 genome is predicted to result in the truncation of seven C-terminal amino-acids in an ABC transporter. The last two at positions 1,245,071 and 1,560,092 are predicted to truncate their associated coding DNA sequence (CDS) in two smaller CDS of similar size, with the loss of 49 and 21 intersticial amino acids respectively. Importantly position 1,245,071 is located within the *prmA* gene (Fig 5). This is particularly interesting since *prmA* was previously shown to have a major impact on virulence in model species *F*. *novicida* [16]. The *prmA* gene in both strains 120 and A-678 is intact and identical to the FSC147 version. Also, we did not find mutations in this gene in any subsp. *mediasiatica* MII strain from our collection (n = 38) when we were assembling WGS reads of these strains on the *prmA* gene of the reference strain FSC147 template.

**Fig 6:**
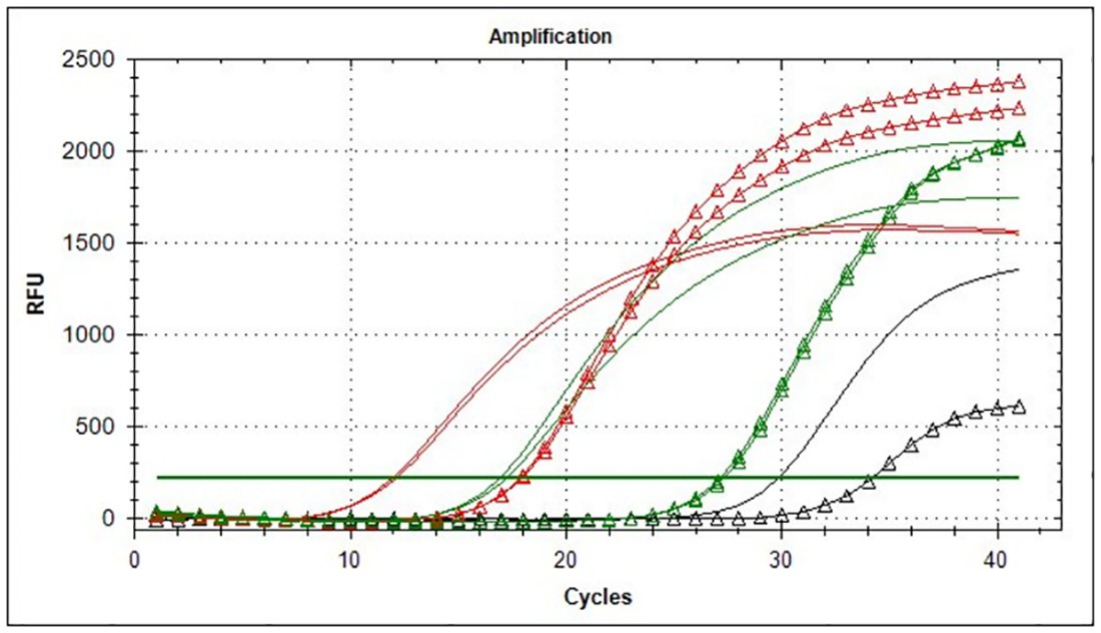
Comparison of the expression levels of the iglC and 16S genes in strains 15NIIEG and 60(B)57A by RT-PCR. Amplification curves of strain 15NIIEG are colored red, Amplification curves of strain 60(B)57A are colored green. The *iglC* gene mRNA amplification curves are indicated by triangles.

To confirm this effect on virulence we compared the expression levels of the *iglC* gene and the *16S* gene in two strains—15NIIEG and 60(B)57A using RT-PCR method after mRNA isolation and reverse transcription. For comparison, the strains were grown for 38 hours on FT agar. We found that the difference in Cq of these genes in strain 15NIIEG is approximately 5 cycles, and in strain 60(B)57A—10 cycles. Moreover, the *iglC* amplification curves for this strain are as close as possible to the negative control curve (nonspecific fluorescence). Apparently they are ahead of the control due to residual impurities of genomic DNA (Fig 6).

## Discussion

Strain 60(B)57, representing subsp. *mediasiatica* group MIII appears to be a spontaneous avirulent strain with immunogenic potential. The virulence of this strain is low enough so that the infectious process does not fully develop but it persists in the host organism long enough to elicit an immune response. We are not aware of previous reports of a spontaneously avirulent *F*. *tularensis* strain. It is curious that this strain belongs to the least studied subspecies, *mediasiatica*, and is currently the unique representative of lineage MIII.

The loss of virulence is predicted to result from the nonsense mutation within the *prmA* gene. The *prmA* gene (also called *qseB* and FTL0552) encoding an orphan response regulator, affects the expression of numerous *Francisella* genes, including those within the pathogenicity island [16–19]. Inactivation of this gene in *F*. *novicida* (which was used as a model for *F*. *tularensis* due to its virulence in mice) resulted in a dramatic decrease in the ability of the mutant strain to survive in macrophages and an absence of virulence in mice. All *F*. *novicida* Δ*prmA*-infected mice survived more than 9 weeks postinfection at all doses up to the highest tested, 1 × 10^8^ CFU [16]. The *F*. *novicida ΔprmA* mutant has been shown to be an effective live vaccine against the homologous challenge. All mice infected with this strain survived subsequent *F*. *novicida* infection challenge even at the highest dose given, 10^8^ CFU intranasally. The induced protection was not efficient against challenge with *F*. *tularensis* subsp. *tularensis* SCHU S4 [16]. Similarly the deletion of *prmA* in the vaccine strain *F*. *tularensis* subsp. *holarctica* LVS led to decrease in the rate of extracellular growth, loss of the ability to replicate in macrophages, decrease in persistence time in the mouse body, and decrease in virulence for mice [20]. Mice that survived infection with the mutant strain were partially protected from intranasal challenge with the SCHU S4 strain 30 days after vaccination [20, 21]. The behaviour of strain 60(B)57, described here, is reminiscent of the described Δ*prmA* mutants. It is avirulent in mice, and protects against homologous challenge with a virulent strain of the same subspecies (*mediasiatica*) for at least 21 days after vaccination. We cannot directly compare the efficiency of heterologous protection of strain 60(B)57 with the results published for Δ*prmA* mutants, as we have significantly increased the time interval between infection and challenge. With an increase in this interval to 90 days, the protection of strain 60(B)57 for mice practically disappears, regardless of the subspecies of the infecting strain. Thus, the 90 days "infection-challenge" time interval we chose might be too long for the mice model. In the guinea pig model, strain 60(B)57 did not protect against challenge with the highly virulent strain SCHU S4 ninety days after infection, but did protect against challenge with strains of the *holarctica* and *mediasiatica* subspecies. Summing up, we can say that the 60(B)57 strain has some immunising properties, but with a much lower efficiency than the existing 15NIIEG vaccine in terms of protection. Therefore, the consideration of subsp *mediasiatica ΔprmA* strains as a hypothetical replacement for the 15NIIEG or LVS strains seems hardly reasonable. The only advantage of strain 60(B)57 seems to be a reduced reactogenicity and high safety of use due to low virulence. With extreme caution, we can assume that such strains might be worth considering when there is a need to vaccinate people who are contraindicated in vaccination with the existing vaccine, for example, due to age or immune status. However, this assumption requires further careful verification [22].

Importantly, the nonsense mutation in the *prmA* gene is not the only mutation distinguishing strain 60(B)57 from other *mediasiatica* strains. Two other nonsense mutations were detected, in addition to tens of missense mutations and a few genome rearrangements. Consequently, although the *prmA* mutation is an excellent putative explanation for the observed avirulence of the strain, we have not provided the formal proof of its implication. This would require the complementation of the strain with a non-mutated allele. However the particular status of *F*. *tularensis*, a category A select agent with potential to be weaponized, forbids such genetic manipulations.

The spontaneous occurrence of an avirulent *F*. *tularensis* strain has not previously been reported to our knowledge. Two scenarios can be proposed. The first is a loss of virulence in nature, prior to the isolation of the strain. The ecology of subsp. *mediasiatica* is currently not well understood. There are no recorded cases of human infection with this subspecies, even in an endemic area (in Altai). The vast majority of subsp. *mediasiatica* strains were isolated from ticks *Haemaphysalis concinna*, *Dermacentor silvarum* and *Ixodes persulcatus* [7]. We are aware of only one strain isolated from wild rodents. So although capable of infecting warm-blooded animals, subsp. *mediasiatica* might naturally exist primarily as an endosymbiont, and spread among ticks by horizontal and/or vertical transmission. In this case, PrmA, which is important for the development of infection in a mammal, may not be necessary for survival in the tick, and inactivation of the corresponding gene might not lead to the death of the strain, allowing it to continue to exist as an endosymbiont. In this case, a decrease in the expression of genes necessary for infecting mammals may even be beneficial for the microorganism, increasing its growth rate in the tick. However, this scenario would predict a more widespread inactivation of *prmA* in subsp. *mediasiatica*, which is not the case.

According to the second scenario strain 60(B)57 lost its virulence during long-term keeping in laboratories and repeated re-sowings. Considering that active work with this strain was not carried out either at the State Research Center for Applied Microbiology and Biotechnology or at the Rostov-on-Don Antiplague Institute, the mutation could have occurred between 1960 and 1986, when this strain was stored in KSCQZD. We have no way of knowing if the virulence of strain 60(B)57 was determined during this time. Given current uncertainties, this second scenario represents the most parsimonious hypothesis.

Clearly, the isolation of additional strains from lineage MIII or others, still unknown *mediasiatica* lineages will be needed to answer this question and contribute to a better understanding of the biology, phylogeny and evolution of subsp. *mediasiatica* within *F*. *tularensis* in general.

## Supporting information

S1 Fig Dynamics of body weight of mice infected with strains 60(B)57 and 60(B)57A. (TIF)

S1 Table Genome assembly accession numbers. (XLSX)

S2 Table List of identified non-synonymous mutations of strain 60(B)57A (compared to the FSC147 genome, accession GCA_000018925). (XLSX)
