# Supplementary figures and images for: Avirulence of a spontaneous Francisella tularensis subsp. mediasiatica prmA mutant
Source: PLoS One. 2024 Jun 18;19(6):e0305569. doi: 10.1371/journal.pone.0305569 (PMC11185464; doi:10.1371/journal.pone.0305569)

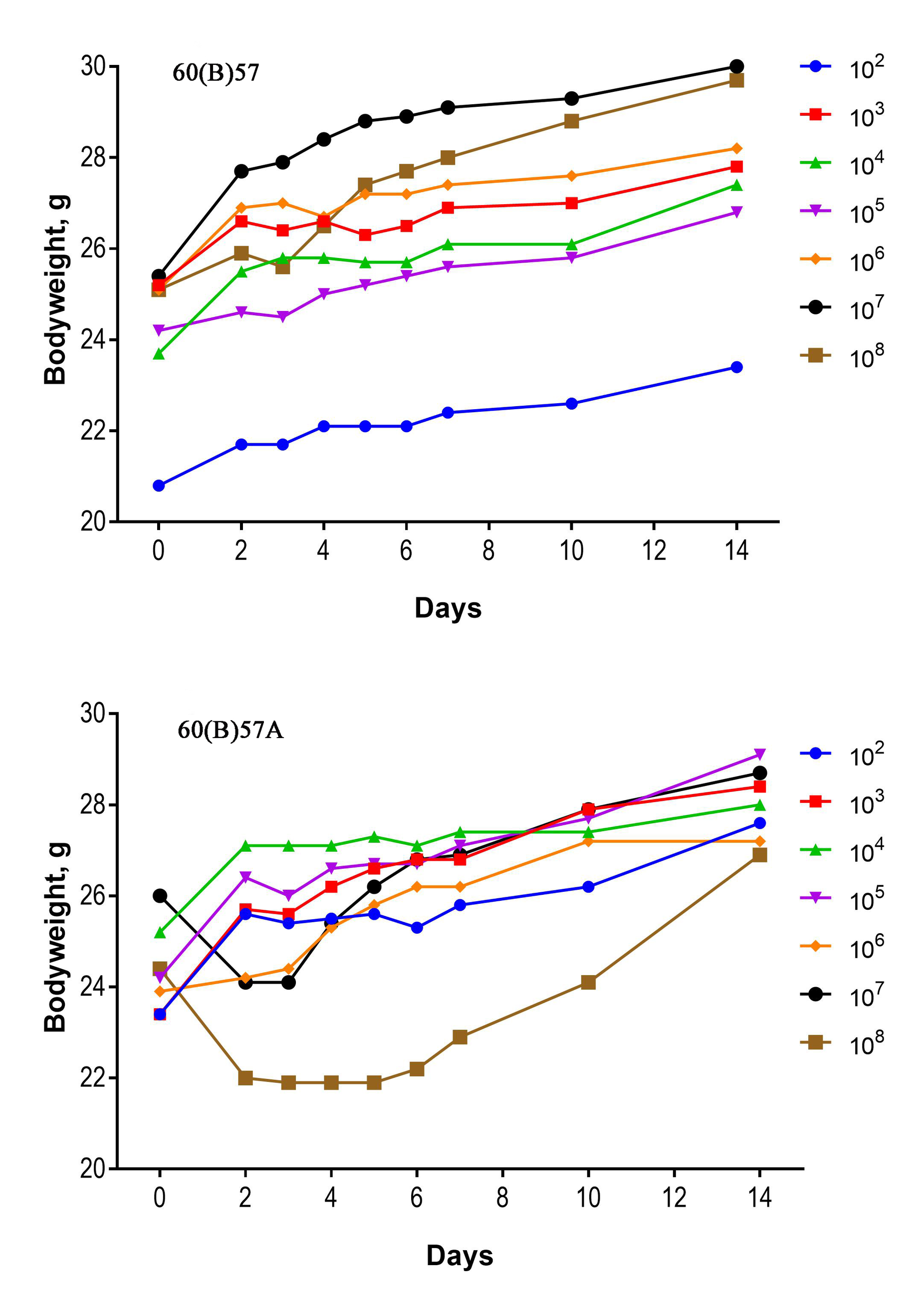

Supplement: S1 Fig — (TIF) [file pone.0305569.s001.tif]
